# Our New York Letter

**Published:** 1889-02

**Authors:** 


### Correspondence.

### OUR NEW YORK LETTER.

TYPHOID FEVER—THE WHITECHAPEL MURDERS DISCUSSED BY THE MEDICO-LEGAL SOCIETY—TROUBLE IN THE UNIVERSITY COLLEGE.

*To the Editors Atlanta Medical and Surgical Journal :*

The subject of typhoid fever was recently discussed before the section on public health of the Academy of Medicine ; the paper of the evening being read by Dr. Cyrus Edson, chief of the Bureau of Contagious Diseases. He regarded this disease as nearly always due to polluted water, milk, ice, or meat, and believed that the germs entered the body through the mouth Wells, he said, were especially dangerous, and safe ones were very scarce. He spoke of the epidemics due to water pollution in North Carolina and Plymouth, Penn., where 1,300 cases occurred. Dr. Fordyce Barker had told him of an instance where 22 cases of typhoid were caused at a popular resort, because the people used ice which had been cut on a lake near by. Milkmen had been known to spread the fever by washing milk cans at infected wells. Dr. Edson believed that digital contagion was possible with typhoid fever, the same as it was with cholera, and that through carelessness, one might carry on his hands the germs and so deposit them on door knobs and bannisters. He had investigated 146 cases occurring in this city and found that 72 were out of town during the preceding 30 days, and of these 29 had been to localities were typhoid was known to exist. Six cases occurred in one house where the drinking water was found to be occasionally contaminated by the contents of the vaults.

Four boys who bathed in the river near sewer outlets were taken down with the fever. He recommended as safety measures care in the use of ice, boiling of milk, and cleanliness of the hands before eating. He said the health department saw that all typhoid cases were visited and disinfectants were used. The Doctor then incidentally read the poem, which had been composed by Mr. James C. Bayles, President of the Health Board, the "Old Oaken Bucket revised and edited by a Sanitarian." (See the January number).

In the discussion of the paper the speakers agreed with the main points set forth by Dr. Edson.

At the last meeting of the Society of Medical Jurisprudence and State Medicine, Mr. Austin Abbott, a lawyer, read a paper on the "Whitechapel Murders and Criminal Lunacy." This mysterious murderer, he said, was skillful and quick, but was not necessarily a surgeon, or a resident of Whitechapel. Even if captured the question of his mental condition would prove very interesting. Man, he said, in the past was naturally brutal, and why should there be any more reason to suspect disease in this murderer than in his ancestors. He probably selected Whitechapel because the victims would mostly be friendless, and this locality would be less dangerous for him. Besides, disease is quite common among the women of this quarter. We would naturally think, he said, that the barbarous pleasure in mutilating his victims could not exist in the same person with the sexual passion, at the same time. One is inconsistent with the activity of the other, and their co-existence showed a deranged mind.

Dr. E. C. Spitzka cited numerous crimes similar, but not approximating the one under discussion. Admitting the murderer had written he had 20 more to kill, it showed real intention, and the non-existence of periodical insanity. Dr. Spitzka caused quite a sensation by stating that "indeed there are stranger freaks of history than in finding the Whitechapel murderer among us now." The subject was also discussed by several other speakers.

During the past few weeks there has been a good deal of trouble at the University Medical College, between the faculty and the students, arising from the alleged neglect of the faculty to appreciate, as the students considered proper, the services and ability of a very popular professor. Dr. Wright, the professor of surgery, who has been in poor health, recently resigned his position, and Professor Lewis A. Stimson succeeded to the same, thus leaving a vacancy in the chair of anatomy. Professor Faneuil D. Weisse, who is a widely known authority on anatomy and is professor of practical and surgical anatomy in the college, volunteered to fill the chair of anatomy during the rest of the term, free of charge. But Dr. Stimson agreed to fill both chairs of surgery and anatomy, and received the faculty's consent to appoint Dr. Woolsey, a graduate of the College of Physicians and Surgeons, as a substitute. Dr. Weisse has taught anatomy for 25 years, and as the college declined to accept his voluntary services, he resigned his position. The students thereupon took his part and demanded that he be appointed to the chair of anatomy, threatening to leave the college in a body if their request was not granted. The students held meetings, and resolutions of a threatening nature were drawn up, but the faculty have remained firm. Dr. Woolsey has already begun to lecture, but at his first appearance 20 students marched out. Three of the ringleaders have been suspended, and although there is still talk of the student's calling a mass-meeting and making a continued fight against the faculty, it is generally believed that at present the worst is over. A. F.

New York, January 8, 1889.

